# Arthrite septique à Proteus mirabilis

**DOI:** 10.11604/pamj.2017.26.197.12271

**Published:** 2017-04-04

**Authors:** Mohammed Sbiti, Bahia Bouhamidi, Lhoussaine Louzi

**Affiliations:** 1 Service de Microbiologie, Hôpital Militaire Moulay Ismail Meknès, Maroc; 2 Faculté de Médecine et de Pharmacie, Université Mohammed Ben Abdellah, Fès, Maroc; 3 Service de Médecine, Hôpital Régional Mohamed VMeknès, Maroc; 4 Faculté de Médecine et de Pharmacie, Université Mohammed V, Souissi-Rabat, Maroc

**Keywords:** Arthrite septique, Proteus mirabilis, facteurs de risqué, Septic arthritis, Proteus mirabilis, risk factors

## Abstract

L'arthrite septique aigue est une pathologie peu fréquente mais grevée d'un pronostic lourd en termes de mortalité et morbidité. Nous rapportons un cas d'arthrite septique à Proteus mirabilis survenue de façon spontanée chez un patient de 61 ansprésentant un diabète compliqué,associée à des hémoculturespositives et des cultures positives du liquide articulaire. L'évolution était favorable grâce au diagnostic précoce et à l'institution d'une antibiothérapie adéquate. L'arthrite septique à Proteus Mirabilis est rare, ce qui nous a incité à revoir dans la littérature des séries d'arthrites à pyogènes incluant Proteus mirabilis portant sur les facteurs de risque, la pathogénie, le traitement et l'évolution de ces pathologies. Le diagnostic est avant tout microbiologique, la ponction articulaire précoce est réalisée avant toute antibiothérapie, l'examen direct, la culture et l'antibiogramme qui va guider le choix d'une antibiothérapie. L'arthrite septique est une urgence diagnostique et thérapeutique, la prise en charge précoce de cette pathologie permet une guérison sans séquelles.

## Abstract (en)

Acute septic arthritis is rare. It is associated with poor prognosis in terms of mortality and morbidity. We report the case of a 61-year old patient with spontaneous Proteus mirabilis septic arthritis. He suffered from complicated diabetes associated with positive blood cultures and synovial fluid cultures. Patient’s evolution was favorable thanks to early diagnosis and initiation of adequate antibiotic therapy. Proteus mirabilis septic arthritis is rare. On that basis we conducted a literature review of cases of Proteus mirabilis pyogenic arthritis to highlight the risk factors, pathogenesis, treatment and evolution of these diseases. Diagnosis is commonly based on microbiological analysis, early articular puncture biopsy is performed before the initiation of antibiotic treatment, direct examination, culture and antibiogram which are useful as guidance for antibiotic therapy. Septic arthritis is a diagnostic and therapeutic emergency; early management of this disease allows total healing without after-effects.

## Introduction

Dans la pratique médicale de tous les jours, il n'estpas rare de voir arriver en urgence un patient avec une ou plusieurs articulations chaudes et gonflées. Plusieurs diagnostics peuvent être évoqués, parmi lesquels l'arthriteseptique à pyogènes qui représente l'une des situations les plus alarmantes. La prise en charge d'une arthrite septique est une urgence, il doit être le plus rapide possible afin d'éviter la destruction irréversible de l'articulation. Le diagnostic repose sur un faisceau d'arguments cliniques bien décrit et doivent être confirmés rapidement par l'étude cytobactériologique de la ponction articulaire, qui reste l'examen diagnostique clef pour la mise en évidence de l'agent pathogène. Nous rapportons ici une rare observation d'arthrite septique à Proteus mirabilis chez un patient diabétique.

## Patient et observation

Il s'agit d'un homme de 61, s'est présentéau service des urgences de l'hôpital régional Mohamed V à Meknès pour un gonflement et douloureux du genou gauche évoluant depuis cinq jours associé à une fièvre à 38,7°C et des frissons. Ce patient avait comme antécédents, un diabètecompliqué de rétinopathie et neuropathie, une hypertension artérielle et une fracture du genou gauche reprises suite à un accident de la voie publique. L'examen clinique montrait un genou gauche augmenté de volume, chaud, douloureux à la palpation avec une impotence fonctionnelle et résistante aux AINS. Il n'y a pas d'adénopathies satellite ni lésions cutanées. Les autres articulations étaient sans anomalies. Le reste de l'examen clinique était sans particularités à part la présence d'une onychomycose avec intertrigo inter orteils. La radiographie du genou gauche ne montrait pas d'anomalies et l'examen tomodensitométrique (TDM) de l'extrémité inférieure du fémur objectivait une arthrose et une collection abcédée en logettes des parties molles postérieures du tiers inférieur du fémur. Les examens biologiques ont objectivé un syndrome inflammatoire (VS à 55 mm à la première heure, CRP à 197ng/ml et fibrinogène 5,6 g/l) et un syndrome infectieux avec à l'hémogramme 18200 / mm^3^ de globules blancs avec 88% de polynucléaires neutrophiles. La glycémie à 2,04g/l, urée et créatinine étaient normales. Les hémocultures étaient positives, les sérologies VIH, VHB, VHC étaient négatives. Le bilan immunologique était normal avec un dosage de complément fraction C4 à 0,40 g/l et des facteurs rhumatoïdes à 7,6uI/ml. La ponction du genou a ramené 4,5 ml de liquide épais, très trouble. L'analyse a montré plus de 3.6 10^6^ leucocytes/ mm^3^ avec 90% de polynucléaires neutrophiles et 10% de lymphocytes, avec absence de microcristaux. L'examen direct a permis de déceler la présence d'une flore bactérienne peu abondante faite de coccobacilles Gram négatif. La culture, après 24 heures, incubée à 37°C, a permis d'identifier Proteus Mirabilis par la galerie API 20E. L'antibiogramme objective la détection d'une une pénicillinase de bas niveau présentant une résistance à l'Amoxicilline et à la Ticarcilline associer à une résistance à la Colistine et l'association Sulfamethoxazole-Trimethoprime. Le patient mis sous Ceftriaxone IV, l'évolution clinique était favorable permettant un retour à l'apyréxie en moins de 48 heures et une diminution des douleurs en quelques jours. La CRP se normalisait en dix jours. Ce traitement était maintenu pendant huit jours, puis relayé par fluoroquinolone seule pendant encore trois semaines.

## Discussion

L'arthrite septique n'est pas une pathologie très fréquente. Dans la littérature il est rare de voir des séries de plus de 50 patients. Classiquement, l'incidence des arthrites septiques est faible dans la population générale, de l'ordre de 2-5 cas/100.000 personnes/an [1]. Des causes générales et locales favorisent les arthrites septiques. Les principaux facteurs de risque associés sont l'âge supérieur à 60 ans, le diabète, la polyarthrite rhumatoïde, une intervention chirurgicale récente, l'existence de prothèse ou d'infection cutanée, 12% des patients atteints d'arthrite septique ont un diabète [2, 3]. L'alcoolisme, la toxicomanie, l'insuffisance rénale et l'hémodialyse, une immunodépression (VIH), un traitement corticoïde ou immunosuppresseur sont souvent décrits dans plusieurs études [4]. Globalement le staphylocoque Aureus est le principal agent responsable de l'arthrite septique à tout âge. Il représente 50 à 80% des étiologies. Les streptocoques hémolytiques surtout du groupe A et dans certains cas, du groupe B, C et G sont les responsables dans 10 à 20% des cas [5]. Les bacilles Gram négatif sont en cause dans 10 à 15% des arthrites septiques. Elles sont généralement retrouvées chez les patients avec des tares comme c'est le cas de notre patient [1]. L'arthrite à Proteus mirabilis est rare chez l'adulte. La pauvreté des informations disponibles sur cette infection nous a incité à réaliser une revue de la littérature. Nous avons trouvé quelques articles représentant des séries d'arthrites septiques à pyogènes ne dépassant pas plus de 50 cas. Comme facteur prédisposant, la polyarthrite rhumatoïde figurait dans presque toutes les séries. Les autres facteurs étaient cités aussi: diabète, traitement par immunosuppresseur, infection à VIH [6–15]. Un seul article comportait un cas de sacro-ileite due à Proteus mirabilis était survenu chez un patient sans aucune comorbidité [16]. Proteus mirabilis présente les caractères des Entérobactéries et les caractères de la tribu des Proteae. Les espèces du genre Proteus sont largement répandues dans la nature et elles sont isolées du sol, de l'eau et de l'intestin de l'homme qu'elles colonisent. Proteus mirabilis serait l'espèce bactérienne la plus souvent impliquée dans les infections urinaires. Ilest également responsable d'infections diverses: surinfections des plaies, infections cutanées, infections del'oreille, du tractus respiratoire et plus rarement de septicémies, méningite et d'atteinte articulaire [17]. Concernant l'atteinte articulaire, l'entrée des bactéries dans l'articulation via le synovial richement vasculariséest facilitée par l'absence de membrane basale. Les germes peuvent aussi pénétrer dans l'articulation à la suite d'une inoculation directe ou plus rarement, par diffusion à partir d'un foyer adjacent [18]. C'est le cas probablement de notre patient étant donné qu'il avait un intertrigo interorteils mais des prélèvements bactériologiques à ce niveau n'ont pas été réalisés à la recherche d'une porte d'entrée.

Bien que la mortalité en cas d'arthrite septique à pyogènes soit relativement faible (<8%) en moyenne dans les séries de la littérature étudiées, un diagnostic et un traitement précoce sont essentiels pour réduire le risque de complications, notamment de destruction articulaire [4–18] Le germe a été isolé dans presque toutes les cultures de liquide articulaire et / ou dans les hémocultures lors des observations de la littérature et chez notre patient. Il est donc indispensable de réaliser des hémocultures et une ponction articulaire avant l'institution de toute antibiothérapie. La mise en culture du liquide articulaire dans des flacons d'hémoculture (Bactec) améliore les résultats comme le montre l'étude de Von Essen et al portant sur l'analyse de 47 cas d'arthrite septique bactérienne [19]. L'antibiothérapie doit être rapidement mise en route. Dans un premier tempsprobabiliste, elle est ensuite adaptée en fonction des résultats de l'antibiogramme. L'antibiothérapie empirique tient compte de l'histoire et de la présentation clinique et des résultats de l'examen directdu liquide synovial après coloration de Gram. Lorsqu'un germe Gram négatif est soupçonné, les céphalosporines de troisième génération, les aminosides et les fluoroquinolones peuvent être utilisés. Selon la société française d'arthroscopie (SFA), la pénétration synoviale des antibiotiques étant excellente,il n'y a pas d'antibiotiques préférentiels. Il faut seulementprivilégier des bithérapies et des doses élevées. Lorsque l'arthrite septique est confirmée, le traitement doit être adapté à l'antibiogramme, le lavage avec du sérum physiologique vise à décomprimer l'articulation et à évacuer les éléments inflammatoires susceptibles d'aggraver le pronostic articulaire. Le traitement chirurgical est exceptionnellement indiqué [4–20]. Pour la durée de l'antibiothérapie, toujours selon la SFA, il est impossible d'établir une ligne de conduite uniciste et il est conseillé de suivre l'antibiothérapie jusqu'à normalisation des marqueurs biologiques [20].Dans les séries étudiées, la durée du traitement était comprise entre 14 et 55 jours [7–9]. Pour notre patient l'antibiothérapie était suivie jusqu'à normalisation de la CRP et elle n'a pas dépassé trois semaines. L'évolution était favorable. Cependant nous n'avons pas pu évaluer les séquelles dans les différentes séries, notamment la limitation de la mobilité articulaire.

## Conclusion

L'arthrite à Proteus mirabilis est une infection grave qui peut compromettre le pronostic vital et fonctionnel. Comme pour toute arthrite septique, il s'agit d'une urgence thérapeutique mais surtout diagnostique et tout retard rejaillit sur la qualité des résultats.La ponction est le geste essentiel, il ne faut pas hésiter à la pratiquer au moindre doute. Une antibiothérapie probabiliste doit être démarrée selon les résultats de l'examen direct. Elle sera ajustée en fonction de l'antibiogramme.
